# Transcriptomic pan‐cancer analysis using rank‐based Bayesian inference

**DOI:** 10.1002/1878-0261.13354

**Published:** 2023-01-23

**Authors:** Valeria Vitelli, Thomas Fleischer, Jørgen Ankill, Elja Arjas, Arnoldo Frigessi, Vessela N. Kristensen, Manuela Zucknick

**Affiliations:** ^1^ Oslo Centre for Biostatistics and Epidemiology University of Oslo Norway; ^2^ Department of Cancer Genetics, Institute for Cancer Research Oslo University Hospital Norway; ^3^ Department of Mathematics and Statistics University of Helsinki Finland; ^4^ Oslo Centre for Biostatistics and Epidemiology Oslo University Hospital Norway; ^5^ Department of Medical Genetics Clinic for Laboratory Medicine Oslo University Hospital Norway; ^6^ Institute for Clinical Medicine Faculty of Medicine University of Oslo Norway

**Keywords:** Bayes Mallows model, cluster analysis, pan‐cancer, robust statistics, subgroup analysis, transcriptomics

## Abstract

The analysis of whole genomes of pan‐cancer data sets provides a challenge for researchers, and we contribute to the literature concerning the identification of robust subgroups with clear biological interpretation. Specifically, we tackle this unsupervised problem via a novel rank‐based Bayesian clustering method. The advantages of our method are the integration and quantification of all uncertainties related to both the input data and the model, the probabilistic interpretation of final results to allow straightforward assessment of the stability of clusters leading to reliable conclusions, and the transparent biological interpretation of the identified clusters since each cluster is characterized by its top‐ranked genomic features. We applied our method to RNA‐seq data from cancer samples from 12 tumor types from the Cancer Genome Atlas. We identified a robust clustering that mostly reflects tissue of origin but also includes pan‐cancer clusters. Importantly, we identified three pan‐squamous clusters composed of a mix of lung squamous cell carcinoma, head and neck squamous carcinoma, and bladder cancer, with different biological functions over‐represented in the top genes that characterize the three clusters. We also found two novel subtypes of kidney cancer that show different prognosis, and we reproduced known subtypes of breast cancer. Taken together, our method allows the identification of robust and biologically meaningful clusters of pan‐cancer samples.

## Graphical abstract

We propose subgroup identification for pan‐cancer studies via novel rank‐based Bayesian clustering, illustrated on RNA‐seq samples from 12 tumor types. The method provides probabilistic assessments of cluster stability and transparent biological interpretation of identified clusters through their top‐ranked genes. We identify robust tissue‐of‐origin clusters but also pan‐cancer clusters including three pan‐squamous clusters with different biological functions over‐represented in top genes.

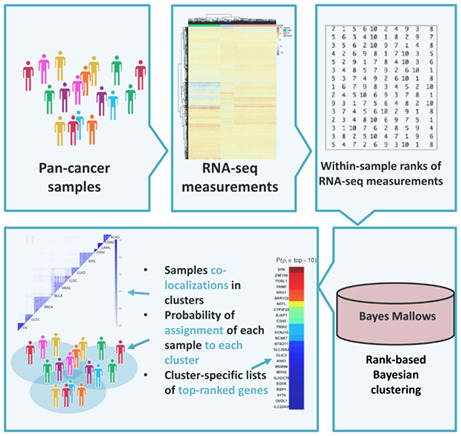

**Abbreviations**
ASCAT: allele‐specific copy number of tumors
BLCA: bladder cancer
BRCA: breast cancer
COAD: colon adenocarcinoma
ER: estrogen receptor
FE: fold enrichment
GBM: glioblastoma
GSEA: Gene set enrichment analysis
HNSC: head and neck squamous cell carcinoma
IQR: interquartile range
KIRC: renal clear cell carcinoma
LAML: acute myeloid leukemia
LUSC: lung squamous cell carcinoma
MCMC: Markov Chain Monte Carlo
OV: ovarian cancer
Q1: first quartile
Q3: third quartile
READ: rectum adenocarcinoma
SFE: selected functional events
SNF: similarity network fusion
TCGA: the Cancer Genome Atlas
UCEC: endometrial cancer

## 1 Introduction

Whole‐genome multi‐omics profiling of tumors has brought unprecedented information and an increased understanding of the characteristics of cancer, allowing the comprehensive investigation of the underlying DNA (mutations, copy number alterations, and epigenetic alterations) and of the phenotype as represented by gene expression profiling and protein profiling. However, the analysis and interpretation of these data are still a challenge. Pan‐cancer molecular studies aim to identify key molecular characteristics that distinguish subtypes of cancer and allow exploring differences and commonalities of tumors originating from different tissues. Hence, emerging knowledge may highlight key cancer‐driving alterations and elucidate the difference between alterations stemming from the cell of origin and alterations stemming from environmental or otherwise external influence. Ciriello et al. [1] performed a molecular analysis of 3299 tumors from 12 cancer types and identified around 500 putative cancer‐driving molecular alterations (selected functional events; SFE) representing copy number alterations, mutations, and epigenetic alterations. They showed that tumors could be classified as driven by either mutations or copy numbers, and that this difference may reflect different oncogenic processes. More recently, more comprehensive pan‐cancer analyses have been performed, including approximately 10 000 tumors from 33 cancer types, using genome‐wide copy number alterations, DNA methylation, mRNA and miRNA, and protein profiles [2]. Integrative multi‐omics clustering of tumors showed that most tumors clustered according to tissue‐of‐origin, while also identifying pan‐cancer clusters including pan‐gastrointestinal [3], pan‐gynecological [4], pan‐kidney [5], pan‐squamous [6], and cancer stemness features [7].

For the purposes of performing a pan‐cancer analysis, clustering methods need to be capable of handling heterogeneous data sources across different tissues. Moreover, fully probabilistic (Bayesian) methods may help to interpret detected subgroups, to allow the integration and propagation of data and model uncertainties into the final results, and to be able to draw probabilistic conclusions that are useful for biological interpretation. A typical drawback of Bayesian methods, and the reason why their use in large‐scale integrative genomics has often been limited, is the heavy computational burden associated with such procedures.

We propose to use a Bayesian rank‐based approach [8], which is both robust and computationally efficient. The use of rankings instead of the actual continuous measurements has already been established as a successful approach to data normalization [9, 10], and it has recently started emerging as a powerful tool for results interpretation [11, 12, 13]. However, to the best of our knowledge, model‐based approaches for rank data have not been proposed in the literature for analyzing‐omics data, neither for the purpose of unsupervised learning nor for prediction. Similarly to the often preferred rank‐based nonparametric alternatives to parametric statistical tests (e.g., the Mann–Whitney *U* test as an alternative to the *t*‐test), we expect the use of a statistical model for rankings to add robustness to the analysis. In addition, the use of ranks eases model assumptions regarding the distributions of the data and allows the joint analysis of heterogeneous data from different tissue types, since ranks are insensitive to scale heterogeneity.

Our rank‐based Bayesian method allows for (a) fully probabilistic uncertainty propagation via prior and modeling choices, (b) data integration from heterogeneous sources, and (c) joint analysis of RNA‐seq data for thousands of tumor samples and genomic features. Here, we apply the method to a pan‐cancer cohort comprising 2617 samples from 12 cancer types from the Cancer Genome Atlas (TCGA). We identify 16 distinct and robust clusters (named RankClusters) and confirm that the tissue of origin is a strong determinant of the transcriptional programs behind each cluster. In addition to confirming known breast cancer subtypes, with this method, we identify three biologically distinct pan‐squamous clusters and two novel subgroups of kidney cancer with different survival prognosis. We demonstrate the precision and robustness of our rank‐based unsupervised model, together with the computational feasibility and usefulness of the Bayesian approach, e.g., for providing easy interpretation of the results, on a previously analyzed benchmark pan‐cancer dataset.

## 2 Materials and methods

### 2.1 Patient data

In this manuscript we reexamined the ‘Pan‐Cancer‐12’ tumor set of 3299 patients, which were also analyzed by Ciriello et al. [1], representing 12 diverse cancer types from TCGA [14]. Pre‐processed RNA sequencing data (Synapse ID syn1715755) were available for a subset of *N* = 2617 of these patients, and the data were downloaded from the corresponding Synapse.org repository (DOI: 10.7303/syn2468297). For computational reasons, and to focus on genes already known to be important for cancer, we selected all gene expression variables, which correspond to genes that are affected by any of the SFEs identified in Ciriello et al. [1]. Gene expression variables with more than 50% missing values were removed. All other missing data were imputed by k‐nearest‐neighbor averaging [15]. The final data set has 1247 gene expression variables associated with SFEs. The study was conducted in accordance with the principles of the Declaration of Helsinki. Analysis of TCGA pan‐cancer data is approved by the Norwegian Regional Ethical Committee with REK number 2016/433. The samples in the original reports were collected with written informed consent from each subject [1, 2].

### 2.2 Principles of the Bayesian statistical analysis

The aim of the statistical analysis is to perform clustering of the samples. The employed clustering method is based on ranks: RNA‐seq gene measurements are ordered for each sample from the largest (rank 1) to the smallest (rank *n*), where *n* indicates the total number of genes in the data set (*n* = 1247). Only the RNA‐seq rank among genes within each sample is used in the analysis. Moving from the original measurement scale to a rank scale can be beneficial, especially when the objective is to jointly analyze samples originating from different tumors: Different tissues might produce data on different scales, which are not necessarily linked to each other linearly, RNA‐seq rankings (instead of absolute values) can allow a more meaningful joint analysis since they are insensitive to scale heterogeneity. Moreover, being able to easily use multiple datasets jointly in one single analysis can lead to more powerful inference.

The clustering method is based on Bayesian inference, to properly integrate measurement errors and model uncertainty into the final cluster landscape. Furthermore, this allows for drawing probabilistic conclusions that are useful in the biological interpretation (a primer to Bayesian inference is provided in Box 1; see also the textbook [16], particularly Section 2.9). The Bayes Mallows model [8] is one of only a few rank‐based Bayesian methods available for clustering purposes, and it shows modeling advantages as compared to the available alternatives [17]. Moreover, the associated r package bayesmallows v0.1.0 [18] has computational advantages over the competitors, as it can handle the large dimensions of the data set at hand.

Box 1 **A primer on Bayesian inference**

In the classical approach to statistics (the so‐called *frequentist* paradigm) the model parameters represent long‐term frequencies, and they are definite, fixed numbers, even though unknown. The aim of the statistical analysis is to provide their estimate from the data at hand. However, such an approach does not take into account any previous information that might make certain parameter values more likely, even before observing the current set of data. An alternative inferential framework, named *Bayesian* paradigm from the central role played by the Bayes theorem, provides instead this possibility thanks to a quite radical change of perspective. In the Bayesian approach, model parameters are random variables, whose distribution ‘*a posteriori*’ is the target of inference. Inferential efforts are not any longer focused on a single unknown value of the model parameters, but a variety of values is allowed, each with its own associated uncertainty. Probability distributions are therefore used to express our knowledge about the parameters even before observing the data, in the so‐called *prior* distributions. Data are then used to update this knowledge via the likelihood, thus obtaining a new parameter distribution (the *posterior*), which can be described and used in different ways to summarize the analysis results. In conclusion: ‘The Bayesian paradigm is a practical approach where prior and posterior distributions are used as models of our knowledge before and after collecting some data and making an observation. It is particularly useful for integrating or combining information from different sources’ [16]; Section 2.9.

To schematically describe the main steps in a Bayesian analysis, we first model the data with a probability distribution, $P\left(\text{data}|\theta \right)$: This is called likelihood because it should be a ‘reasonably good’ mathematical description of the data‐generating process. With θ one generally indicates the set of model parameters, i.e., parameters in the assumed data distribution, which are the purpose of the analysis. Next, one needs to decide a reasonable probabilistic distribution for θ: $P\left(\theta \right)$ is called prior, because it represents an initial guess about parameters; priors can be noninformative, in case no relevant information is available. Finally, thanks to Bayes' Theorem:

$$P\left(\theta |\text{data}\right)\propto P\left(\text{data}|\theta \right)\times P\left(\theta \right).$$

One can perform inference about θ based on their *a posteriori* (i.e., *after the data*) distribution $P\left(\theta |\text{data}\right)$. The posterior distribution is therefore a combination of prior and likelihood, in the sense that the *a priori* distribution of model parameters is *updated* with data information, as expressed in the likelihood. This has some important implications: The posterior of model parameters is generally close to the likelihood for big data; when instead data are low‐dimensional or sparse, clinical knowledge as embedded in the prior becomes very relevant. For a thorough introduction to Bayesian inference, we suggest to consult the first chapters in Bernardo and Smith [42]. For a very quick glimpse on the typical steps of a Bayesian analysis, we refer to the recent primer on Nature Reviews [43]. A more hands‐on introduction to Bayesian thinking can be found in the book by Holmes and Huber [16], in Section 2.9.

### 2.3 The rank‐based clustering method

Let $R_{\mathrm{ij}}$ be the rank given to gene $A_{i}$ by sample *j*, for *i* = 1, … *n* and *j* = 1, … *N* ($R_{j}$ in vectorial notation). A Mallows model [19] is a distance‐based probability distribution for $R_{j}$ defined on the ranking space (the permutation space $P_{n}$ of dimension *n*), which is very similar to a generalized Gaussian distribution for continuous data. If we assume that the *N* observed rankings follow the Mallows model, the data likelihood then takes the form

$$P\left(R_{1},\ldots ,R_{N}|\alpha ,\rho \right)=Z_{n}{\left(\alpha ,\rho \right)}^{-N}\exp\left\{\frac{-\alpha }{n}\sum_{j=1}^{N}d\left(R_{j},\rho \right)\right\}\prod_{j=1}^{N}1_{P_{n}}\left(R_{j}\right),$$

The Mallows model depends on two parameters: a location parameter ρ, called consensus ranking, which is a ranking describing the center of the distribution (the analogous of the mean for a Gaussian distribution), and a scale parameter α, which is a scalar describing its variation (analogous to the precision). Therefore, if the Mallows model is assumed to describe the distribution of the observed rankings of genes in the sample, the probability of observing each patient's specific ranking of genes will be larger the closer the ranking is to the consensus ranking, i.e., the location parameter, quite the same as for normally distributed data in the continuous case. The closeness is measured by the chosen distance between rankings, $d\left(.,.\right)$, and weighted by the scale parameter α. In other words, given a set of patients, each providing an observed ranking of genes, the Mallows consensus ranking estimates (with uncertainty) the gene ranking that best describes the agreement within the set (see [8] for more technical details on the model). The chosen distance between rankings also influences the computational burden of the procedure, and we used the Spearman footrule (equivalent to the L1 norm for permutations) for its efficiency [18] and robustness to extreme data [17]. Evidently, the agreement of all samples around a single consensus gene ranking cannot be reached, since cancer samples are expected to be quite heterogeneous in their molecular characteristics, and thus, the scope of the analysis is to estimate possible grouping structures of the samples around several gene rankings, by means of a clustering method. A model‐based clustering method making use of a finite mixture of Mallows distributions with *C* components (clusters) is here used to jointly estimate the groups and the group‐specific consensus gene rankings. Note that ‘group’ and ‘cluster’, as well as ‘grouping’ and ‘clustering’, will be used interchangeably throughout the paper. Each cluster *c* = 1, … *C* gets a specific location $\rho_{c}$ and scale $a_{c}$ parameter, and each sample is assigned to a cluster via the latent variables $z_{1},\ldots z_{N}\in \left\{1,\ldots C\right\}$. In the clustering framework, the model likelihood then takes the form

$$\begin{array}{l} P\left(R_{1},\ldots ,R_{N}|{\left\{a_{c}\right\}}_{c=1}^{C};{\left\{\rho_{c}\right\}}_{c=1}^{C};z_{1},\ldots z_{N}\right)\\ \;=\prod_{j=1}^{N}\frac{1_{P_{n}}\left(R_{j}\right)}{Z_{n}\left(a_{z_{j}},\rho_{z_{j}}\right)}\exp\left\{\frac{-a_{z_{j}}}{n}\sum_{j=1}^{N}d\left(R_{j},\rho_{z_{j}}\right)\right\}. \end{array}$$

Bayesian inference is used with uninformative or, when convenient, conjugate priors, to exploit computational shortcuts without imposing too much prior information to the model (see [8] for all model specifications and inferential details). A Metropolis‐Hastings‐based Markov Chain Monte Carlo (MCMC) algorithm is used to estimate the parameters, and convergence is assessed by standard approaches (by a posteriori inspecting the parameter chains and acceptance probabilities). See Fig. S1 for a visual explanation of the Bayesian rank‐based clustering method.

### 2.4 Selection of the number of clusters and cluster dynamics

The clustering algorithm produces, for each pre‐specified number *C*, a mixture distribution of *C* clusters around their respective consensus gene rankings. We propose both a strategy to evaluate the best value for *C*, and a dynamic view on the clustering results across different choices of *C*, which describes the stability of the clustering when varying *C*. Note that, contrary to commonly used hierarchical clustering methods, mixture‐based clustering methods are nonhierarchical. Therefore, the model does not force the grouping structures to remain consistent across different numbers of clusters. This allows for greater flexibility in finding a reasonable posterior‐based partition of the samples for any value of *C*, and removes the serious weakness of the ‘greediness’ of hierarchical clustering methods, which can result in sub‐optimal local solutions.

In order to estimate the value of *C* that best describes the heterogeneity in the data, we compute a measure of cluster fit that is based on the within‐cluster sum of distances of the observed gene rankings from the cluster consensus gene ranking: $\sum_{c=1}^{C}\sum_{j:z_{j}=c}d\left(R_{j},\rho_{c}\right)$. This measure decreases markedly if adding one more cluster provides a better picture of the grouping structure in the data, and remains stable if the addition is not worthwhile. Since we perform Bayesian inference, we can inspect the boxplots of the posterior distribution of this measure as a function of *C*. This plot helps to select the most suited value for *C*, usually corresponding to an ‘elbow’ in the boxplots, i.e., a change in trend from a marked decrease in the measure to a stabilization for larger values of *C*. Note that in Bayesian clustering, the cluster assignments are also probabilistic. Hence, to be able to compute the within‐cluster sum of distances of the observed rankings, we need to assign each sample to a unique cluster. The easiest and most reasonable way to do so is a majority vote on each samples' assignment probabilities to the *C* clusters.

Concerning the dynamics of the clustering results, since our mixture‐based Bayesian clustering approach is nonhierarchical, if samples are often assigned to the same group when varying *C*, it is a sign that the estimated groupings describe stable biologically meaningful structures. We measure this stability by computing the proportion of times each pair of samples was clustered together when varying *C* in a reasonable range of values (from a minimal $C_{m}$ being the smallest mixture giving trustable results, to a maximal $C_{M}$ being the largest mixture given the computational constraints). We thus obtain a co‐localization N×N dimensional matrix *S* whose elements take the form:

$$S\left(i,j\right)=\frac{\sum_{C=C_{m}}^{C_{M}}1\left({\bar{z}}_{i}^{C}={\bar{z}}_{j}^{C}\right)}{C_{M}-C_{m}+1},\text{for}\;i,j=1,\ldots N$$

where ${\bar{z}}_{i}^{C}$ and ${\bar{z}}_{j}^{C}$ are the majority vote cluster assignments of samples *i* and *j*, respectively, when using a mixture with *C* clusters, and where $C_{m}=10$ and $C_{M}=30$. Values in *S* range from 0 to 1, with 1 indicating pairs of samples that are always grouped together, and 0 indicating pairs that are never grouped together. Therefore, observing a block structure in *S* indicates good stability of the clustering across values of *C*.

### 2.5 Estimation of the cluster‐specific gene lists

A secondary but equally important aim of the analysis is to describe the commonalities in the RNA‐seq measurements of the samples assigned to the same cluster in terms of the estimated cluster consensus gene ranking, i.e., the cluster‐specific ranked list of genes. Each cluster in the mixture model is characterized by the consensus ranking $\rho_{c}$ of the genes, which is estimated with uncertainty by the MCMC algorithm. Therefore, in order to inspect the gene‐wise commonalities of samples belonging to the same group, it is sufficient to summarize the marginal posterior distribution of the corresponding cluster‐specific consensus ranking $\rho_{c}$, for c=1,…C.

Specifically, for each cluster c=1,…C, we compute a cluster‐specific list of genes by ranking the genes according to their posterior probability of being top‐ranked in the cluster‐specific consensus ranking, with ‘top‐k’‐ranked indicating a rank not larger than *k*, for *k* ranging from 10 to 100. Specifically, this entails computing, for each cluster *c* and each specific value of *k*, the marginal posterior probability: $P\left.\left(\rho_{\mathrm{ic}}<k|R_{1},\ldots ,R_{N};{\left\{a_{c}\right\}}_{c=1}^{C};z_{1},\ldots ,z_{N}\right)\right.$ for all genes i=1,…n. Ranking such probabilities allows for obtaining several cluster‐specific gene lists, which can be used for different purposes. For instance, we observe that very few genes show a significant posterior probability of being top‐10 ranked, and thus choosing *k* = 10 results in stable lists of few genes, which can easily be studied comprehensively. They represent sparse and easily interpretable molecular characterizations of the clusters, which still capture the most important characteristic features of each cluster. On the other hand, we find that larger numbers of genes show a large posterior probability of being top‐100 ranked, and the resulting larger gene lists can be assumed to be a robust choice of relevant genes, which can for example be used in a subsequent gene set analysis.

### 2.6 Gene set enrichment analysis

Gene set enrichment analysis (gsea) was performed using the hallmark (H) and gene ontology (C5) gene set collections obtained from the Molecular Signatures Database v7.1 [20]. *P*‐values for over‐representation were calculated by one‐sided hypergeometric testing (R function *phyper*), where each gene list of interest was tested for association to each gene set. *P*‐values were corrected for multiple testing using the Benjamini–Hochberg procedure (R function *P.adjust*), and the enrichment was considered significant if the corrected *P*‐value was smaller than 0.05. To minimize the bias caused by the 1247 SFE‐associated genes, this gene set was used as background when the gsea was performed.

### 2.7 Survival analysis

For survival analyses, we downloaded the curated survival data for TCGA [21], and Kaplan–Meier analyses and log‐rank tests for progression‐free survival were performed using the r package *survminer* v0.4.9 [22].

### 2.8 Differential gene expression analysis

Differential gene expression analysis was performed using the nonparametric Mann–Whitney *U* test (R function *wilcox.test*) for all genes. The difference between groups was considered statistically significant if the two‐sided FDR‐corrected *P*‐value according to the method of Benjamini and Hochberg [23] was smaller than 0.05.

### 2.9 Bayes Mallows clustering with random gene set

To assess the robustness of the Bayes Mallows method and the importance of the choice of gene set, we also performed the Bayes Mallows clustering on a random selection of 1247 genes (see Table S1), and compared the distributions of samples across clusters.

### 2.10 Unsupervised hierarchical clustering

To compare the Bayes Mallows clustering to traditional clustering methods, we performed unsupervised hierarchical clustering using both the SFEs‐associated gene set and the random gene set. The heatmaps and dendrograms were produced with the r package *pheatmap* v. 1.0.12 [24], and the clustering was performed using Euclidean distance and Ward linkage (R function *hclust* method *ward.D2*).

### 2.11 Software for statistical analysis

All statistical analyses were performed using the r software version 4.2.0 [25].

## 3 Results

### 3.1 Rank‐based Bayesian clustering of pan‐cancer samples

**Fig. 1:**
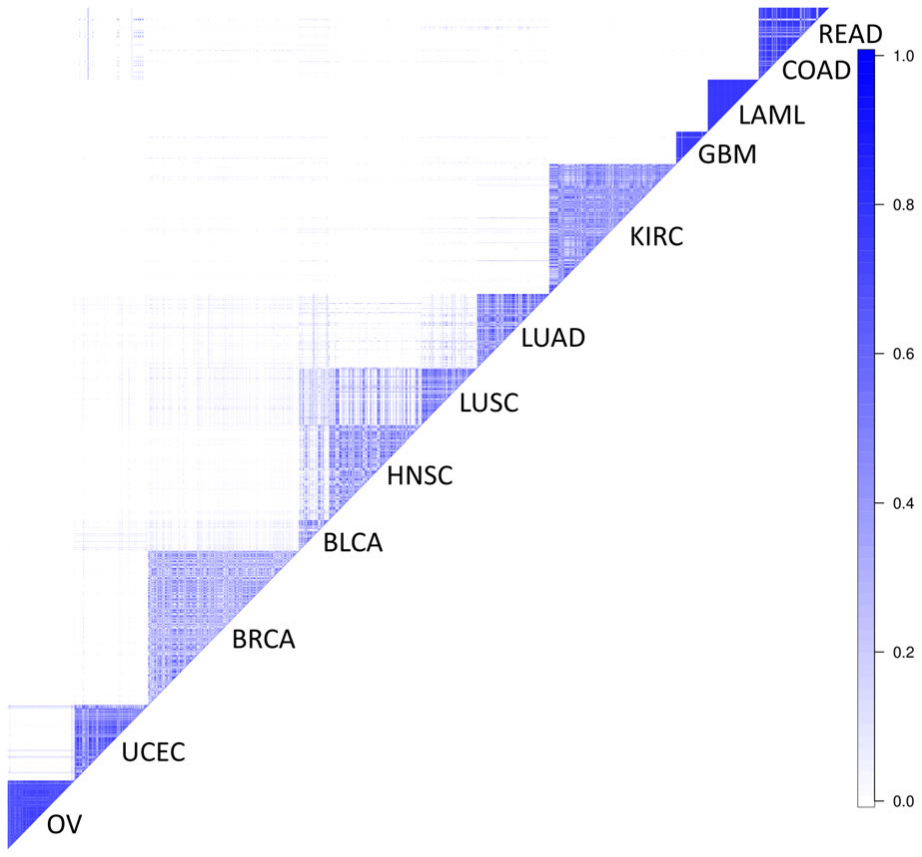
Stability of cluster assignments, as measured by the co‐localization matrix *S*. The frequencies of the samples' co‐localizations in clusters obtained with number of clusters (*C*) varying from 10 to 30 (from white to blue, values from 0 to 1). BLCA, bladder cancer; BRCA, breast cancer; COAD, colon adenocarcinoma; GBM, glioblastoma; HNSC, head and neck squamous cell carcinoma; KIRC, renal clear cell carcinoma; LAML, acute myeloid leukemia; LUSC, lung squamous cell carcinoma; OV, ovarian cancer; READ, rectum adenocarcinoma; UCEC, Endometrial cancer.

We applied the Bayes Mallows approach to the ‘Pan‐Cancer‐12’ tumor set of 2617 patients interrogating the gene expression of 1247 genes representing selected functional (cancer‐driving) events [1] (and named SFEs‐associated gene set, see the ‘Patient data’ paragraph in Materials and methods), with the aim to identify robust clusters of cancer samples both between and within cancer types. Figure 1 depicts the matrix *S* obtained by counting the samples' co‐localizations in the same cluster for the groupings obtained with *C* (the number of groups) varying from 10 to 30. Note that we chose to not consider grouping structures obtained for *C* < 10 since they clearly under‐represent the variation in the data, and thus do not provide a reasonably meaningful clustering of the samples. Figure 1 shows that the cluster dynamics is quite stable across different choices of *C* even if the hierarchy is not enforced by the mixture‐based clustering procedure: Indeed, most values in *S* are either close to 0 or 1, indicating that the clusters represent a biologically meaningful grouping, not influenced by the model choices and model adequacy. Samples from the same tissue are often seen stably clustered together (especially for OV, GBM, and LAML), and a pan‐cancer cluster composed of BLCA, HNSC, and LUSC appears for many values of *C*. Some cancer types are occasionally found together, indicating that they cluster together at lower values of *C* (UCEC and COAD/READ, and BRCA and LUAD).

**Fig. 2:**
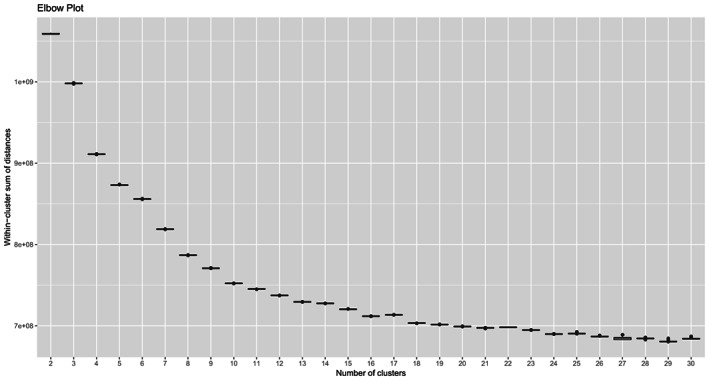
Elbow plot for optimal selection of number of clusters (*C*). Boxplots of the posterior distributions of the within‐cluster sum of distances of the observed rankings from the cluster location parameters, for any choice of the number of clusters *C* from 2 to 30. We sampled 1000 realizations from the posterior distribution to build each boxplot. In each boxplot, the middle horizontal line corresponds to the posterior median, the box margins correspond to the posterior Q1 and Q3, and vertical lines (i.e., the boxplot whiskers) stretch to the largest (smallest) realization that is larger (smaller) than Q3 + 1.5*IQR (Q1 − 1.5*IQR). Any realizations falling outside such limits are considered outliers and drawn separately with a point.

To determine the most appropriate number of clusters (*C*) we inspected the boxplots of the posterior distributions of the within‐cluster sum of distances (Fig. 2), which steadily decrease for *C* increasing from 2 to 30. We can notice the last major decrease in this cluster‐fit measure for *C* = 16, which then tends to stabilize for *C* > 16. Therefore, and to also meet a parsimony criterion, 16 clusters seems the best choice for further inspection.

### 3.2 Identification of 16 patient clusters (RankClusters) based on the Bayes Mallows clustering

**Fig. 3:**
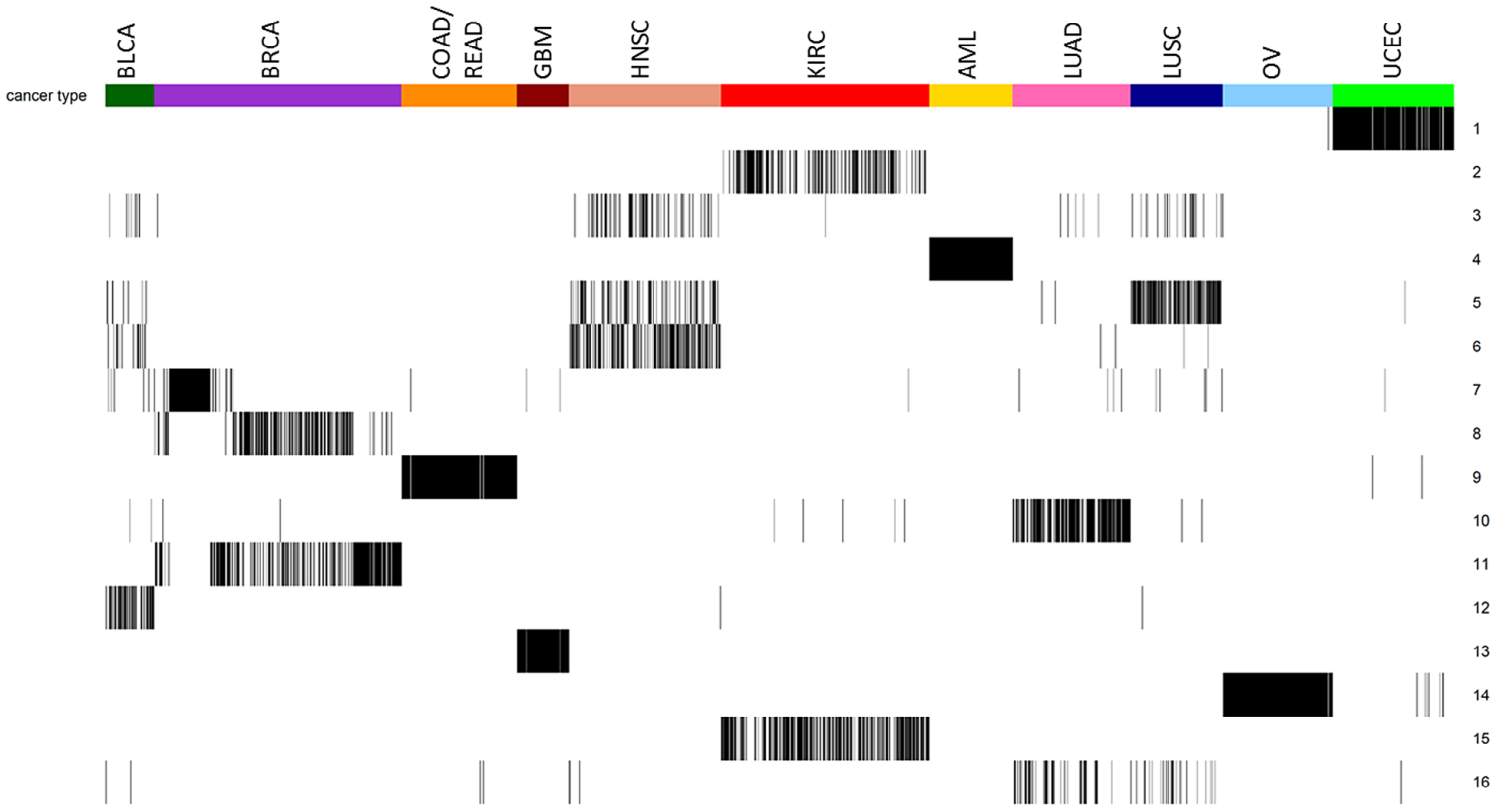
Cluster assignments of tumor samples obtained via Bayes Mallows clustering when fixing the number of clusters (*C*) to 16. Samples are ordered according to the tissue of origin, and the cluster assignment is reported along the rows. BLCA, bladder cancer; BRCA, breast cancer; COAD, colon adenocarcinoma; GBM, glioblastoma; HNSC, head and neck squamous cell carcinoma; KIRC, renal clear cell carcinoma; LAML, acute myeloid leukemia; LUSC, lung squamous cell carcinoma; OV, ovarian cancer; READ, rectum adenocarcinoma; UCEC, Endometrial cancer.

The grouping results obtained for 16 clusters showed a trend that samples cluster according to organ site, confirming that tissue of origin is a strong determinant for global gene expression profiles and phenotypes (Fig. 3). Five cancer types clustered in separate clusters: endometrial cancer (UCEC; RankCluster 1), acute myeloid leukemia (LAML; RankCluster 4), colon and rectum adenocarcinoma (COAD and READ; RankCluster 9), glioblastoma (GBM; RankCluster 13), and ovarian cancer (OV; RankCluster 14). Two cancer types were assigned to two or three clusters, where these clusters still mostly contained one cancer type: breast cancer (BRCA; RankCluster 7, 8, and 11) and renal clear cell carcinoma (KIRC; RankCluster 2 and 15). Finally, we discovered three pan‐cancer clusters of tumors with squamous characteristics (RankCluster 3, 5, and 6; pan‐squamous clusters) containing head and neck squamous cell carcinomas (HNSC), lung squamous cell carcinoma (LUSC), and bladder cancer (BLCA).

To assess how sensitive the method is to the gene set selection, we ran the Bayes Mallows clustering method also on a random selection of 1247 genes. For the random gene set, we chose *C* = 17 (elbow plot; Fig. S2A), and we observed almost perfect overlap between the clustering results obtained when using the SFEs‐associated gene set and the random gene set (see Fig. S2B for the clustering of samples based on the random gene set, and Fig. S2C for the concordance table). We can therefore conclude that Bayes Mallows is robust to a possibly noisy gene set selection. Hierarchical clustering largely recapitulated results from the Bayes Mallows RankClusters, also mostly identifying clusters representing tissue of origin (Fig. S3A). There was good concordance between the RankCluster assignments and the hierarchical clustering (Fig. S3B), and the clustering was also robust when using the random gene set (Fig. S3C).

### 3.3 Characterization of the 16 RankClusters in terms of top‐ranked genes

**Fig. 4:**
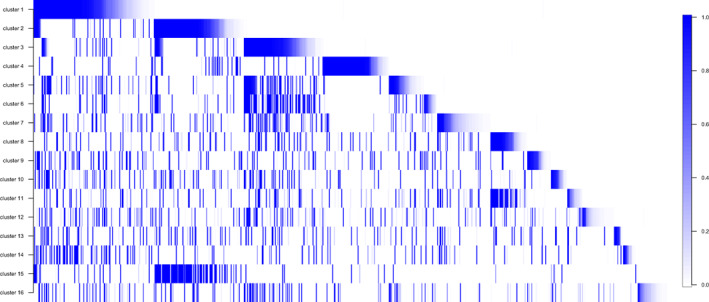
Top genes characterizing the 16 RankClusters, sorted by the probability of being ranked among the top 100. Horizontal axis represents the genes ordered (from left to right) by decreasing probability (only when larger than 0.01), for each consecutive RankCluster (1–16).

An important advantage of the Bayes Mallows method is that it also estimates specific gene rankings characterizing each cluster (as a by‐product of the marginal posterior probability distribution of the cluster‐specific consensus ranking), thus allowing easy investigation of upregulated pathways characterizing clusters. Specifically, each RankCluster can be characterized by the group of genes showing the largest probability of being top‐ranked: In Fig. 4, for example, the genes are ranked according to the probability of being among the top 100 in the cluster consensus ranking, for each of the 16 RankClusters (see Tables S2 and S3 for the rankings and probabilities of genes being top 10 for each RankCluster, and Tables S4 and S5 for the ranking and probabilities of genes being top 100 for each RankCluster). Some RankClusters shared more top genes with other RankClusters than one might expect: RankClusters 3, 5, and 6 (pan‐squamous) share many top genes, as does cluster RankCluster 8 and 11 (BRCA), and RankCluster 2 and 15 (KIRC). Conversely, RankCluster 4 (AML) showed very few shared top genes, much less than on average (Fig. 4). This co‐occurrence of genes in the RankClusters top lists can be quantified: For each RankCluster, we selected the genes having at least 1% probability of being ranked top‐100, and called them ‘top‐ranked genes’. Then, we computed the density of the counts of how many times a top‐ranked gene/(for a given RankCluster) was top‐ranked also for other RankClusters. These cluster‐specific densities can then be tested (via a Kolmogorov–Smirnov goodness‐of‐fit test) to show that the co‐occurrence counts are stochastically larger (or smaller) than expected if due to random variation only. The pan‐squamous RankClusters all showed stochastically larger than average counts (*P*‐value < 0.0001), while RankClusters 4, 8, and 16 all showed co‐occurrence counts stochastically smaller (*P*‐value < 0.0001) than average (see Fig. S4 for a plot of the cluster‐specific densities of the counts of the top‐ranked genes co‐occurrences). These findings support the claim that the pan‐squamous RankClusters show common biological characteristics, while other RankClusters share comparatively fewer genes.

### 3.4 Bayes Mallows clustering identifies three pan‐squamous clusters

RankCluster 3 contained 66 HNSC, 27 LUSC, and eight BLCA samples, RankCluster 5 contained 75 HNSC, 116 LUSC, and eight BLCA samples, and RankCluster 6 contained 150 HNSC, 16 BLCA, and two LUSC samples, illustrating that subgroups of squamous cell carcinomas share more commonalities across the tissue of origin than to other carcinomas of same tissue of origin (Fig. 3).

**Fig. 5:**
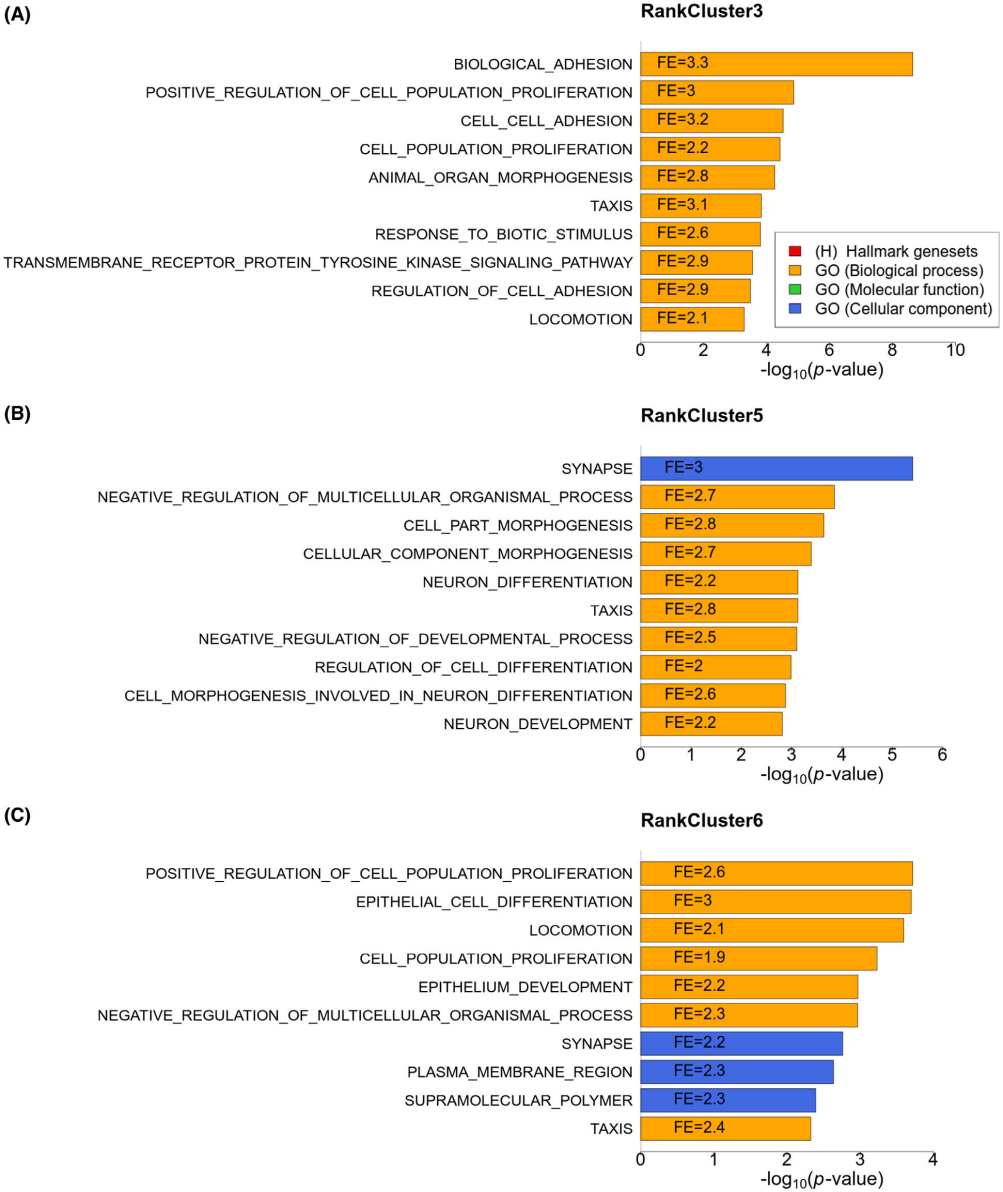
Gene set enrichment analysis (gsea) results of the top‐ranked genes of the three pan‐squamous RankClusters for (A) RankCluster 3, (B) RankCluster 5, and (C) RankCluster 6. *P*‐value of enrichment (hypergeometric test) is shown on the *x*‐axis, and fold enrichment (FE) is shown inside the bars.

To assess the biological mechanisms driving the identified RankClusters we performed a gsea on the top‐ranked genes characterizing each cluster. For this analysis, the top‐ranked genes characterizing a cluster were defined as genes with at least 80% probability of being ranked among the top 100 in the cluster consensus gene ranking. The top‐ranked genes in RankCluster 3 were enriched in several gene sets related to adhesion and proliferation (Fig. 5A); the top‐ranked genes in RankCluster 5 were enriched in gene sets related to morphogenesis and cell differentiation (Fig. 5B); and the top‐ranked genes in RankCluster 6 were enriched in gene sets related to epithelial cell differentiation, proliferation and locomotion (Fig. 5C). Thus, the three pan‐squamous clusters share some biological characteristics (e.g., altered phenotype related to invasiveness), and some are unique to each RankCluster (e.g., adhesion for RankCluster 3, morphogenesis for RankCluster 5 and proliferation for RankCluster 6). The gsea results indicate that the three RankClusters could represent different disease mechanisms shared across the tissue of origin, and also that squamous cancers share certain biological characteristics. The pan‐squamous RankClusters were not significantly associated with the prognosis of patients (Fig. S5). gsea of the top‐ranked genes of the RankClusters identified using the random gene set selection provided few or no significantly enriched overlaps with known gene sets.

Pan‐cancer clusters of squamous cell carcinoma have been discovered previously [2] where the majority of squamous cell carcinomas were assigned to four integrative clusters (iC10, iC20, iC25, and iC27). Although there was a significant association between the two classifications (Table 1A; chi‐square test *P*‐value = 9.02e‐20), it was clear that the two clustering methods did not capture precisely the same variation. Hierarchical clustering of squamous cell carcinomas using mRNA expression has been reported by Campbell et al. [6], and a quite strong overlap was observed between this classification and the classification reported here (Table 1B; chi‐square test *P*‐value = 1.81e‐86). The mRNA clusters 2 and 3 mostly contain samples, which were clustered into RankClusters 5 and 6, respectively. However, by splitting mRNA clusters 1, 4, and 6, RankCluster captured additional and complementary information.

**Table 1:** Comparison between the pan‐cancer clusters of squamous cell carcinomas obtained via Bayes Mallows and (A) the integrative clusters [2]; (B) the mRNA clusters [6]. Numbers in parentheses are percentages.

A	iC2	iC7	iC10	iC13	iC17	iC20	iC25	iC27
RankCluster 3	1 (0.2)	0 (0)	26 (6.1)	0 (0)	0 (0)	26 (6.1)	14 (3.3)	19 (4.4)
RankCluster 5	2 (0.5)	11 (2.6)	93 (21.7)	3 (0.7)	6 (1.4)	8 (1.9)	24 (5.6)	36 (8.4)
RankCluster 6	0 (0)	3 (0.7)	49 (11.4)	0 (0)	0 (0)	0 (0)	34 (7.9)	74 (17.2)

B	mRNA1	mRNA2	mRNA3	mRNA4	mRNA5	mRNA6
RankCluster 3	26 (5.9)	2 (0.5)	3 (0.7)	13 (3)	0 (0)	45 (10.3)
RankCluster 5	54 (12.3)	96 (21.9)	3 (0.7)	34 (7.7)	3 (0.7)	0 (0)
RankCluster 6	2 (0.5)	6 (1.4)	106 (24.1)	31 (7.1)	0 (0)	15 (3.4)

### 3.5 Novel subtyping of kidney renal clear cell carcinoma

**Fig. 6:**
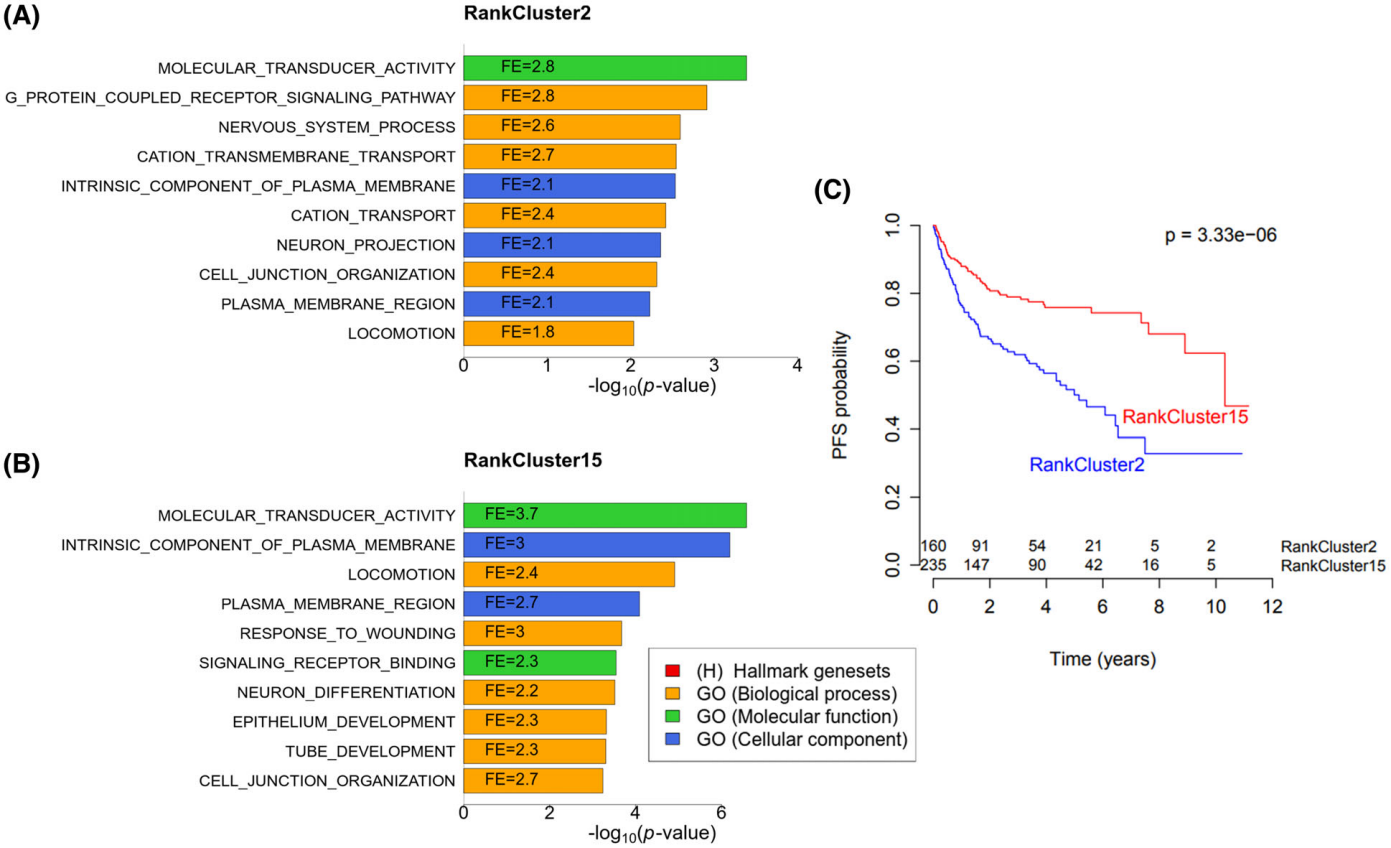
Gene set enrichment analysis (gsea) results and survival analysis of the two renal clear cell carcinoma (KIRC) clusters. gsea *P*‐value of enrichment (hypergeometric test) is shown on the *x*‐axis, and fold enrichment (FE) is shown inside the bars for RankCluster 2 (A) and RankCluster 15 (B). (C) Kaplan–Meier plots of progression‐free survival and corresponding log‐rank test *P*‐value.

RankClusters 2 and 15 contained only KIRC samples: 162 in RankCluster 2 and 235 in RankCluster 15. These clusters shared 66 top‐ranked genes (of 88 and 91, respectively), and gsea showed that the genes in both clusters were enriched in gene sets related to transducer activity and plasma membrane transport (Fig. 6A,B). Interestingly, the subdivisions of the KIRC samples were not dependent on the gene set used for clustering, as the distribution across RankClusters was almost identical when we performed the clustering using the random gene set. To investigate the differences between RankClusters 2 and 15, we performed genome‐wide differential gene expression analysis between samples in the two clusters: 5158 genes (of 15 647) were significantly differentially expressed (Mann–Whitney *U* test; Benjamini–Hochberg FDR‐corrected *P*‐value < 0.05), showing that the two subgroups of KIRC share key biology and simultaneously differ with regard to transcriptional programs. Survival analysis showed that patients in RankCluster 15 had better progression‐free survival compared with patients in RankCluster2 (log‐rank test *P*‐value = 3.33e‐06; Fig. 6C).

KIRC samples in TCGA have previously been characterized and mRNA hierarchical clustering identified four subgroups of KIRC samples [26]. We observe a significant overlap between the RankClusters and the mRNA clusters (chi‐square test *P*‐value = 1.71e‐34), with mRNA cluster 1 containing almost exclusively samples from RankCluster 15. Among the three other mRNA clusters, the samples were quite evenly distributed between RankCluster 2 and 15 (Table 2).

**Table 2:** Comparison between the RankCluster classification of the renal clear cell carcinoma (KIRC) samples and the classification of the same samples reported in TCGA 2013 [26]. Numbers in parentheses are percentages.

	mRNA1	mRNA2	mRNA3	mRNA4
RankCluster 2	3 (0.8)	63 (16.3)	32 (8.3)	64 (16.5)
RankCluster 15	130 (33.6)	24 (6.2)	51 (13.2)	20 (5.2)

### 3.6 RankClustering of breast cancer recapitulates known subgroups

BRCA samples were divided into three clusters: RankClusters 7, 8, and 11 (Fig. 3). When comparing with PAM50 subtypes [27], the estrogen receptor (ER) positive breast tumors (Luminal A and Luminal B) were found in RankClusters 8 and 11, with the majority of Luminal A in RankCluster 8 and the majority of Luminal B in RankCluster 11 (Table 3). Her2‐enriched samples were mostly found in RankCluster 11 with some in RankCluster 7 (Table 3). These results suggest that we identify one less aggressive subgroup (RankCluster 8) and one more aggressive subgroup (RankCluster 11) among the ER‐positive breast tumors. Our results also highlight the uncertainty in the separation into Luminal A and Luminal B [28], since these samples are distributed between RankClusters. Basal‐like (ER‐negative) samples were found in RankCluster 7 (together with some BLCA, GBM, and LUSC). Taken together, these results show that our method identifies known biology and clinical features among breast tumors.

**Table 3:** Comparison between the RankCluster classification and PAM50 subtypes of breast cancer (BRCA) samples [27]. Numbers in parentheses are percentages.

	Luminal A	Luminal B	Her2‐enriched	Basal‐like	Normal‐like
RankCluster 7	0 (0)	0 (0)	10 (1.9)	83 (15.4)	10 (1.9)
RankCluster 8	161 (29.9)	9 (1.7)	2 (0.4)	0 (0)	28 (5.2)
RankCluster 11	91 (16.9)	91 (16.9)	33 (6.1)	0 (0)	21 (3.9)

### 3.7 Tumor purity across RankClusters

To assess whether the identified clusters could be influenced by tumor purity, we compared the allele‐specific copy number of tumors (ASCAT)‐estimated tumor purities [2] across RankClusters (Fig. S6A). We observed clear differences in tumor purity, with RankCluster 4 (LAML) showing high purity (median = 0.8) and RankCluster 3 (pan‐squamous) showing low purity (median = 0.35); indeed, RankCluster3 showed significantly lower tumor purity than all RankClusters except 10 and 16 (Fig. S6B), and this may partly explain the separation of these samples from the other pan‐squamous samples in RankClusters 5 and 6. For the KIRC samples, there was no statistically significant difference in tumor purity between RankCluster 2 and 15 (Fig. S6B); for the BRCA samples, there was no statistically significant difference between RankCluster 7 and 8, while there was a small difference between RankCluster 7/8 and RankCluster 11 (*P*‐values 2.1e‐2 and 4.3e‐4, respectively; Fig. S6B).

## 4 Discussion

We have presented a pan‐cancer analysis performed using a rank‐based Bayesian mixture model, i.e., a clustering method based on the Bayes Mallows model, which allows for drawing probabilistic conclusions. Bayesian inference allows for expressing the uncertainty associated with all results, which is useful in biological interpretation. The use of ranks instead of original measurements gives greater flexibility in combining heterogeneous data sources (across tissues) as pre‐processing to match measurement scales is not required, which in turn allows analyses of combined datasets, e.g., for a pan‐cancer analysis. A parametric mixture‐based model (e.g., Li et al. [29], which was specifically developed for clustering RNA‐seq data by a mixture of negative binomial distributions) would be expected to perform poorly on pan‐cancer data, because of the additional layer of heterogeneity between samples due to different cancer tissues and other study‐specific effects. In addition, the rank‐based approach also avoids other problems associated with the fact that the statistical distribution of RNA‐seq data is challenging. It is highly right‐skewed and heteroscedastic count data, which require sophisticated modeling, i.e., a combination of the use of statistical distributions for over‐dispersed count data (e.g., the negative binomial distribution) with regularization and data transformations that take into account the variance–mean dependence in count data [30, 31].

Another advantage of the Bayes Mallows approach is that the clustering also provides lists of the most important genes characterizing each cluster, thus allowing easy interpretation of identified clusters. Note that another advantage of a fully Bayesian model, which we did not explore further in this paper, is the possibility to embed *a priori* information into the model estimates, for example, by giving higher *a priori* rank to genes that are known to be relevant in cancer.

In concordance with previous reports, we observe that most tumors cluster according to the tissue of origin [2, 32], and we report that heterogeneous cancer types such as BRCA and KIRC are divided between two or more clusters. Importantly, we also identify three pan‐squamous clusters containing LUSC, HNSC, and BLCA where the tumors do not cluster according to the tissue of origin but according to cell morphology. Previous discoveries of pan‐squamous clusters [2, 6] have also been able to subdivide these tumors into novel subtypes. Our work highlights that some tumors are more similar across tissue types than within the same tissue of origin, suggesting that the clinical classification could be re‐examined for improved classification of these patients. Further research should be carried out to assess prognosis and treatment response across tissue of origin.

Our method identified two molecular subtypes of KIRC with very different prognosis and transcriptional programs. The 2013 TCGA paper [26] reports four subtypes of KIRC, which partly overlap with those identified presently; together these reports illustrate the potential benefit of molecular pathology for kidney renal clear cell carcinoma. The results for breast cancer confirm known biological heterogeneity of this cancer type (e.g., all Basal‐like tumors fall in RankCluster 7) [33, 34, 35]. Moreover, our clustering illustrates the known difficulty in separating between Luminal A and Luminal B tumors, and that this separation is likely a continuum (both subtypes are distributed in RankClusters 8 and 11). Her2‐enriched tumors may either be ER‐positive‐like or ER‐negative‐like (i.e., belong to RankClusters 7 or 11).

The application of the Bayes Mallows method to a random subset of genes resulted in a very similar clustering of cancer samples compared with the selection of 1247 gene expression features associated with the SFEs identified in Ciriello et al. [1] (Fig. S2), which illustrates that the clusters are robust and can be reproduced by many different gene sets without prior biological knowledge [36]. However, gsea with the top‐100 ranked gene lists from the clustering with random genes did not result in any statistically significantly enriched gene sets. These results indicate that while the gene set selection is not crucial for cluster assignment, it is clearly important for biological interpretation. One may hypothesize that this is because the clustering identifies transcriptional programs that may, e.g., be determined by the cell of origin; but the biological interpretation will be easier if the selected genes are more studied (i.e., part of more curated pathways and implicated in disease).

The rank‐based Bayesian Mallows clustering method currently presents some weaknesses, which we hope to address in future to bring the model even closer to the needs of integrative genomics applications. The first and most important aspect concerns computational costs: The Bayesian estimation procedure is currently based on an MCMC algorithm, which is known to be inefficient and can be slow, particularly for high‐dimensional applications. The current implementation limits the number of features to be ~ 1000 (like in the current pan‐cancer application) and requires hours to run in such a situation. The average computing time for Bayes Mallows with *C* = 16 was 9 h12’, while mixtures with a smaller (larger) number of groups require slightly less (more) computing time. We are currently working on a Variational Bayes alternative to the MCMC algorithm [37] that samples efficiently from an approximate model; this will significantly speed up computation, as this alternative implementation is scalable in the number of features and of mixture components.

Secondly, the method is limited in how many features (e.g., in this pan‐cancer application, how many genes) it can handle, not only for computational but also for modeling reasons. While it is reasonable to assume that the pre‐selection of 1247 genes associated with the SFEs are all expressed and would rank differently in different clusters, this would not be a reasonable assumption for a genome‐wide data set, because differences across clusters in the top‐ranked genes would affect the likelihood in the same way as differences way down in the ranking would do. A biologically more sound assumption could be that only the genes that are expressed in the tissues of interest are the ones driving cluster differences. The method's limitation to ~ 1000 genes also puts limitations on the biological interpretation of cluster‐associated gene lists, as different selections of genes will give different results in a gsea. We are currently working on a dimension reduction version of the method, which will address this limitation [38].

Finally, it would be very relevant to propose a generalization of the Bayes Mallows method to perform data integration across multiple omics data types (multi‐view clustering). With such a generalization, we would be able to provide a rank‐based clustering of the samples that jointly account for several data views. The gold standard for multi‐view clustering is still snf [39] and iclusterplus [40] for a Bayesian implementation using a joint latent variable model, while alternative methods have also recently been proposed to perform hierarchical data fusion [41]. A Bayes Mallows generalization for multi‐view clustering would transfer the advantages of rank‐based clustering to the multi‐omics clustering task, including more robustness against deviations from parametric model assumptions and to the additional layer of heterogeneity in pan‐cancer studies. This Bayes Mallows generalization would, however, require a dimension reduction and scalable version of the method, both of which are currently under development.

## 5 Conclusion

We have presented the novel use of a Bayesian rank‐based clustering method for the purposes of a pan‐cancer analysis of a gene expression data set from TCGA. As with any other Bayesian approach, the method has the advantage of providing proper uncertainty quantification of all unknowns, together with probabilistic interpretation of all results. This is particularly helpful in the analysis of large molecular databases, to allow straightforward assessment of the stability and reliability of conclusions, and to provide biological characterization and interpretation of the identified clusters. In addition to tissue‐specific clusters, we identified three pan‐squamous clusters composed of a mix of lung squamous cell carcinoma, head and neck squamous carcinoma, and bladder cancer, with different biological functions enriched in the lists of top‐ranked genes. We also identified two subtypes of kidney cancer with very different survival prognosis, and reproduced known subtypes of breast cancer. Taken together, our method allows robust gene expression‐based clustering of pan‐cancer samples, providing novel biologically meaningful insight.

## Conflict of interest

The authors declare no conflict of interest.

## Author contributions

TF, VV, MZ, AF, EA, and VNK conceptualized the manuscript. VV and MZ performed the rank‐based Bayesian cluster analysis. TF and JA performed the additional statistical analysis. TF, VV, and MZ interpreted the data. TF and VV wrote the manuscript with contributions from all other authors. All authors read and approved the final version of the manuscript.

### Peer review

The peer review history for this article is available at https://publons.com/publon/10.1002/1878‐0261.13354.

## Supporting information


**Fig. S1.** Explanation of the Bayesian rank‐based clustering method.
**Fig. S2.** Bayes Mallows clustering method on a random selection of 1247 genes.
**Fig. S3.** Hierarchical clustering and comparison with RankClusters and random gene selection.
**Fig. S4.** Density of the counts of how many times a top‐ranked gene (for a given RankCluster) was top‐ranked also for other RankClusters for genes with at least 1% probability of being ranked top‐100 in each cluster.
**Fig. S5.** Kaplan–Meier plot of progression‐free survival for patients in the three pan‐squamous RankClusters.
**Fig. S6.** Average allele‐specific copy number of tumors (ASCAT)‐estimated tumor purities for the 16 RankClusters.

**Table S1.** Random genes used for clustering.

**Table S2.** Gene rankings (probability of being top 10).

**Table S3.** Probabilities of genes (Table S2) being top 10.

**Table S4.** Gene rankings (probability of being top 100).

**Table S5.** Probabilities of genes (Table S4) being top 100.

## Data Availability

Pre‐processed RNA sequencing data (Synapse ID syn1715755) is available from the Synapse.org repository (DOI: 10.7303/syn2468297).
