# Supplementary material for: Transcriptomic pan‐cancer analysis using rank‐based Bayesian inference
Source: Mol Oncol. 2023 Jan 23;17(4):548–63. doi: 10.1002/1878-0261.13354 (PMC10061294; doi:10.1002/1878-0261.13354)
Supplement: Supplementary file 1 — Fig. S1. Explanation of the Bayesian rank‐based clustering method. Fig. S2. Bayes Mallows clustering method on a random selection of 1247 genes. Fig. S3. Hierarchical clustering and comparison with RankClusters and random gene selection. Fig. S4. Density of the counts of how many times a top‐ranked gene (for a given RankCluster) was top‐ranked also for other RankClusters for genes with at least 1% probability of being ranked top‐100 in each cluster. Fig. S5. Kaplan–Meier plot of progression‐free survival for patients in the three pan‐squamous RankClusters. Fig. S6. Average allele‐specific copy number of tumors (ASCAT)‐estimated tumor purities for the 16 RankClusters. [file MOL2-17-548-s004.pptx]

## Slide 1
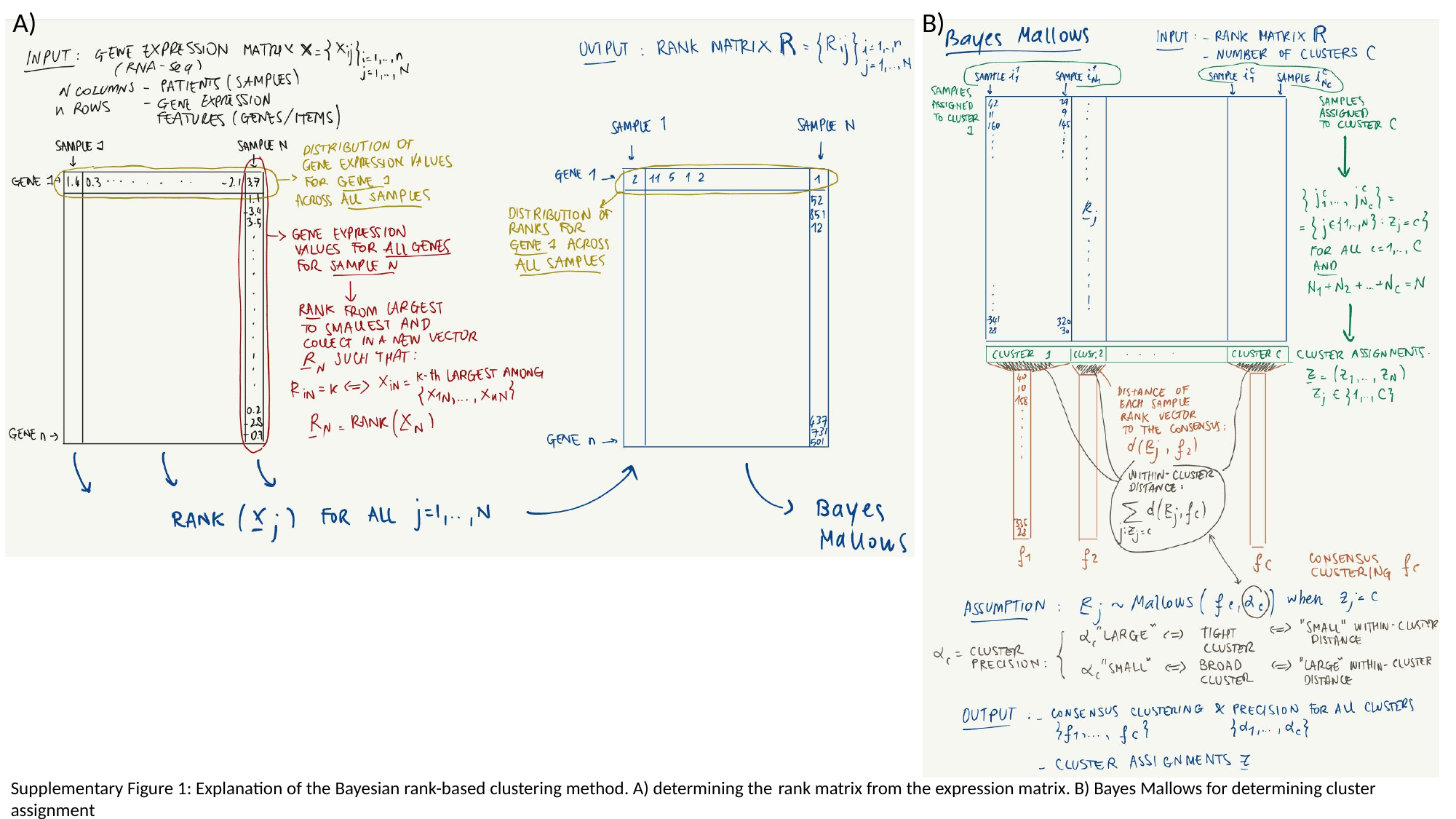

A)
B)
Supplementary Figure 1: Explanation of the Bayesian rank-based clustering method. A) determining the rank matrix from the expression matrix. B) Bayes Mallows for determining cluster assignment

## Slide 2
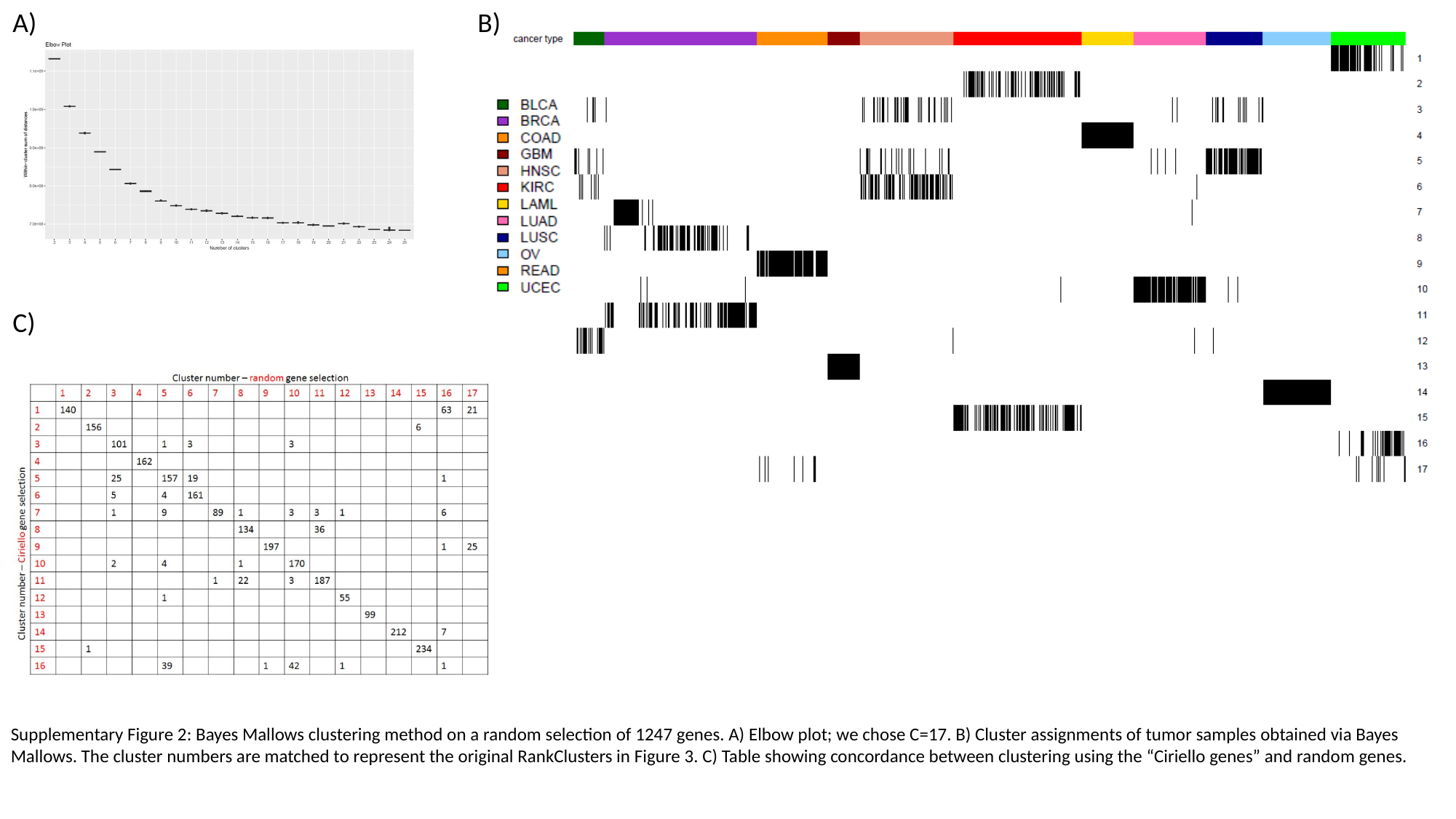

A)
B)
C)
Supplementary Figure 2: Bayes Mallows clustering method on a random selection of 1247 genes. A) Elbow plot; we chose C=17. B) Cluster assignments of tumor samples obtained via Bayes Mallows. The cluster numbers are matched to represent the original RankClusters in Figure 3. C) Table showing concordance between clustering using the “Ciriello genes” and random genes.

## Slide 3
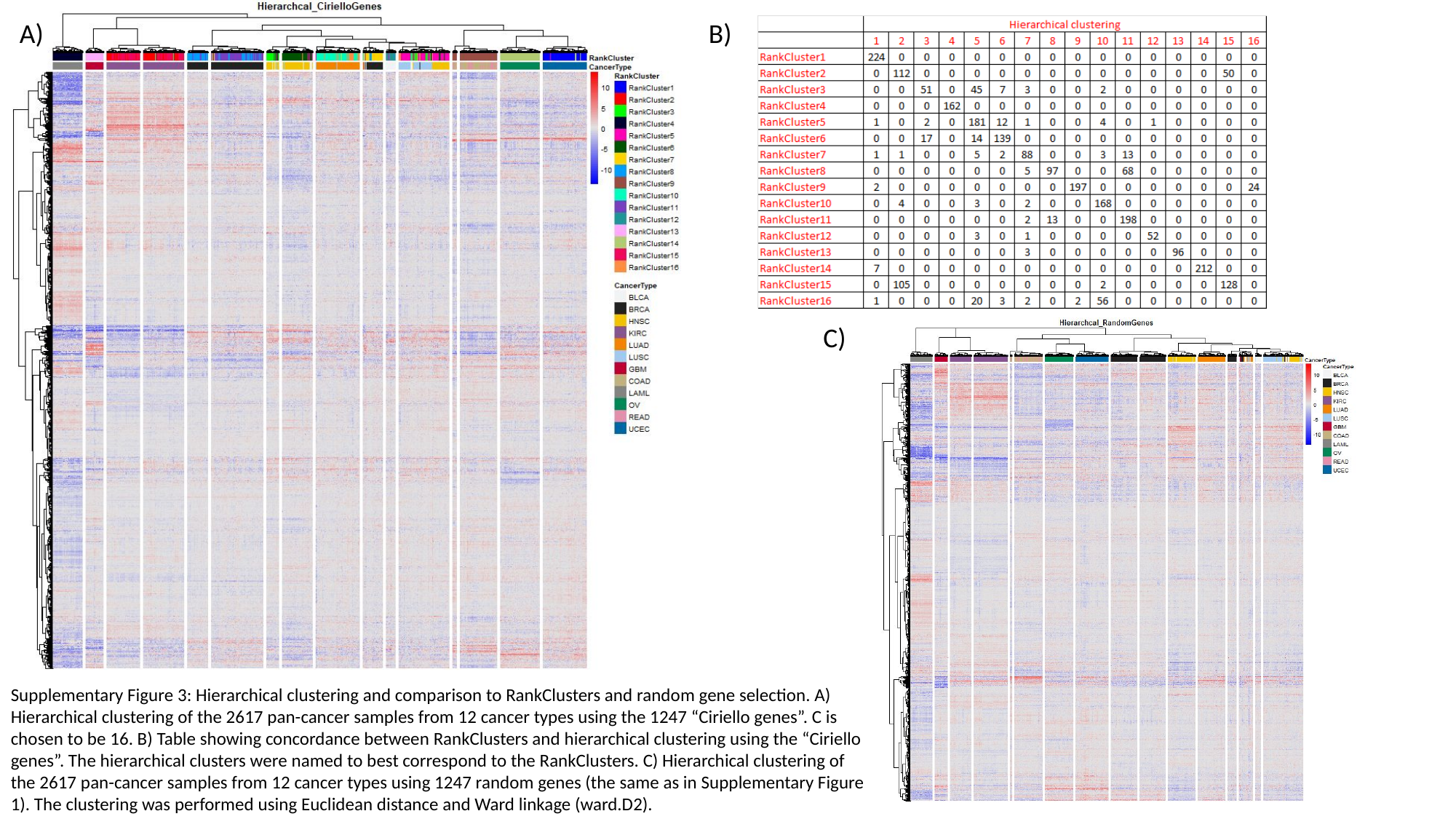

A)
B)
C)
Supplementary Figure 3: Hierarchical clustering and comparison to RankClusters and random gene selection. A) Hierarchical clustering of the 2617 pan-cancer samples from 12 cancer types using the 1247 “Ciriello genes”. C is chosen to be 16. B) Table showing concordance between RankClusters and hierarchical clustering using the “Ciriello genes”. The hierarchical clusters were named to best correspond to the RankClusters. C) Hierarchical clustering of the 2617 pan-cancer samples from 12 cancer types using 1247 random genes (the same as in Supplementary Figure 1). The clustering was performed using Euclidean distance and Ward linkage (ward.D2).

## Slide 4
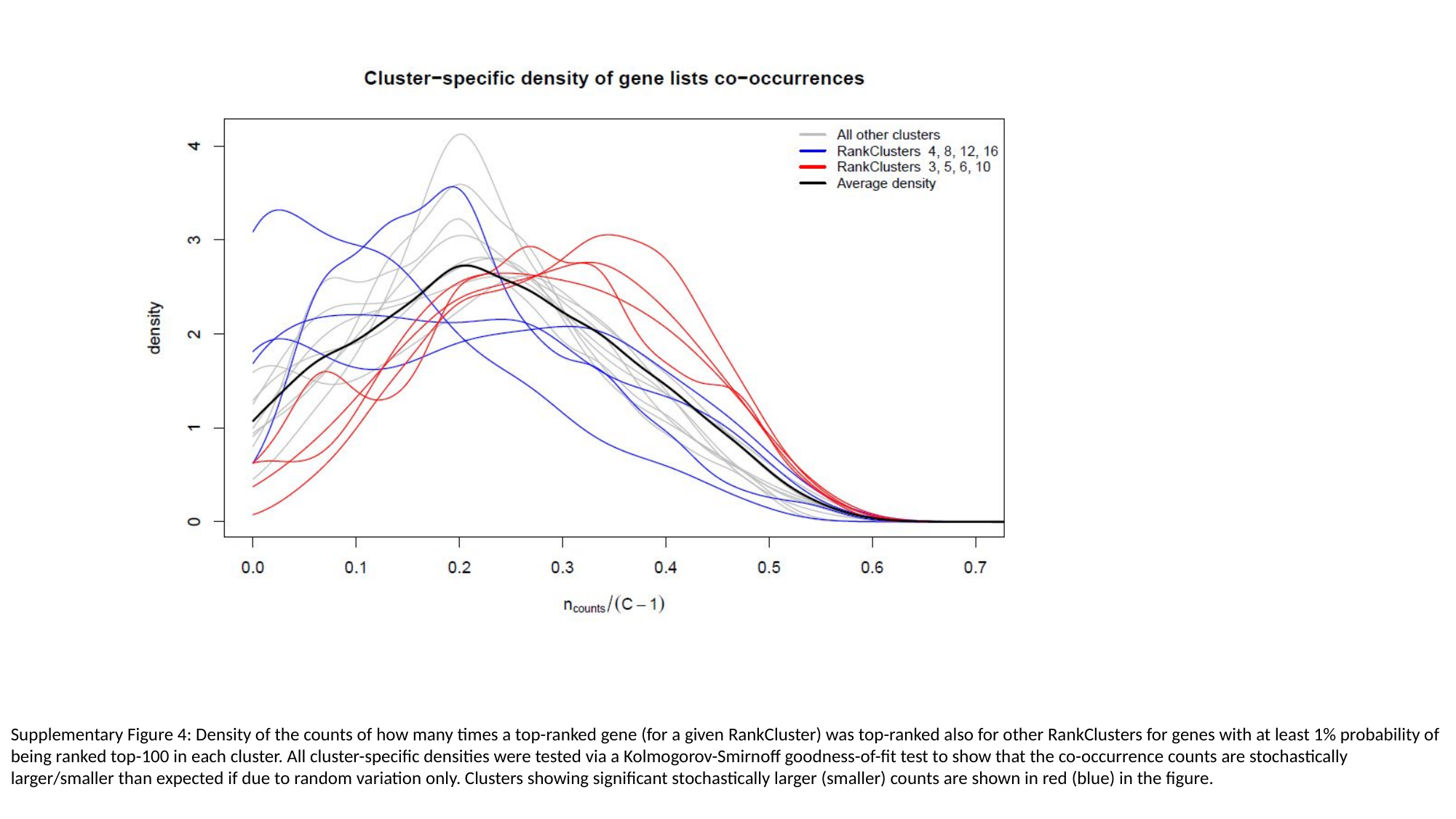

Supplementary Figure 4: Density of the counts of how many times a top-ranked gene (for a given RankCluster) was top-ranked also for other RankClusters for genes with at least 1% probability of being ranked top-100 in each cluster. All cluster-specific densities were tested via a Kolmogorov-Smirnoff goodness-of-fit test to show that the co-occurrence counts are stochastically larger/smaller than expected if due to random variation only. Clusters showing significant stochastically larger (smaller) counts are shown in red (blue) in the figure.

## Slide 5
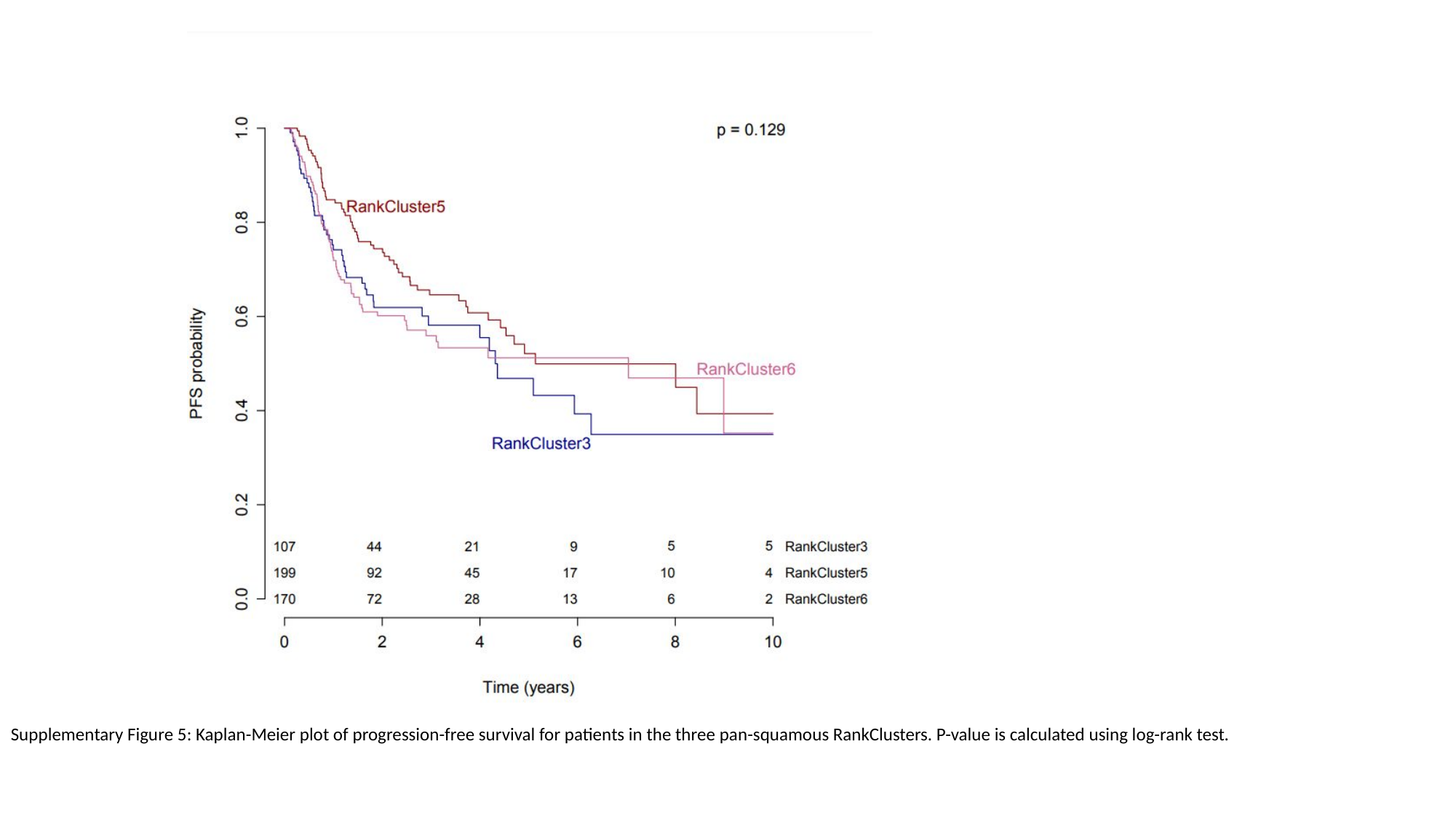

Supplementary Figure 5: Kaplan-Meier plot of progression-free survival for patients in the three pan-squamous RankClusters. P-value is calculated using log-rank test.

## Slide 6
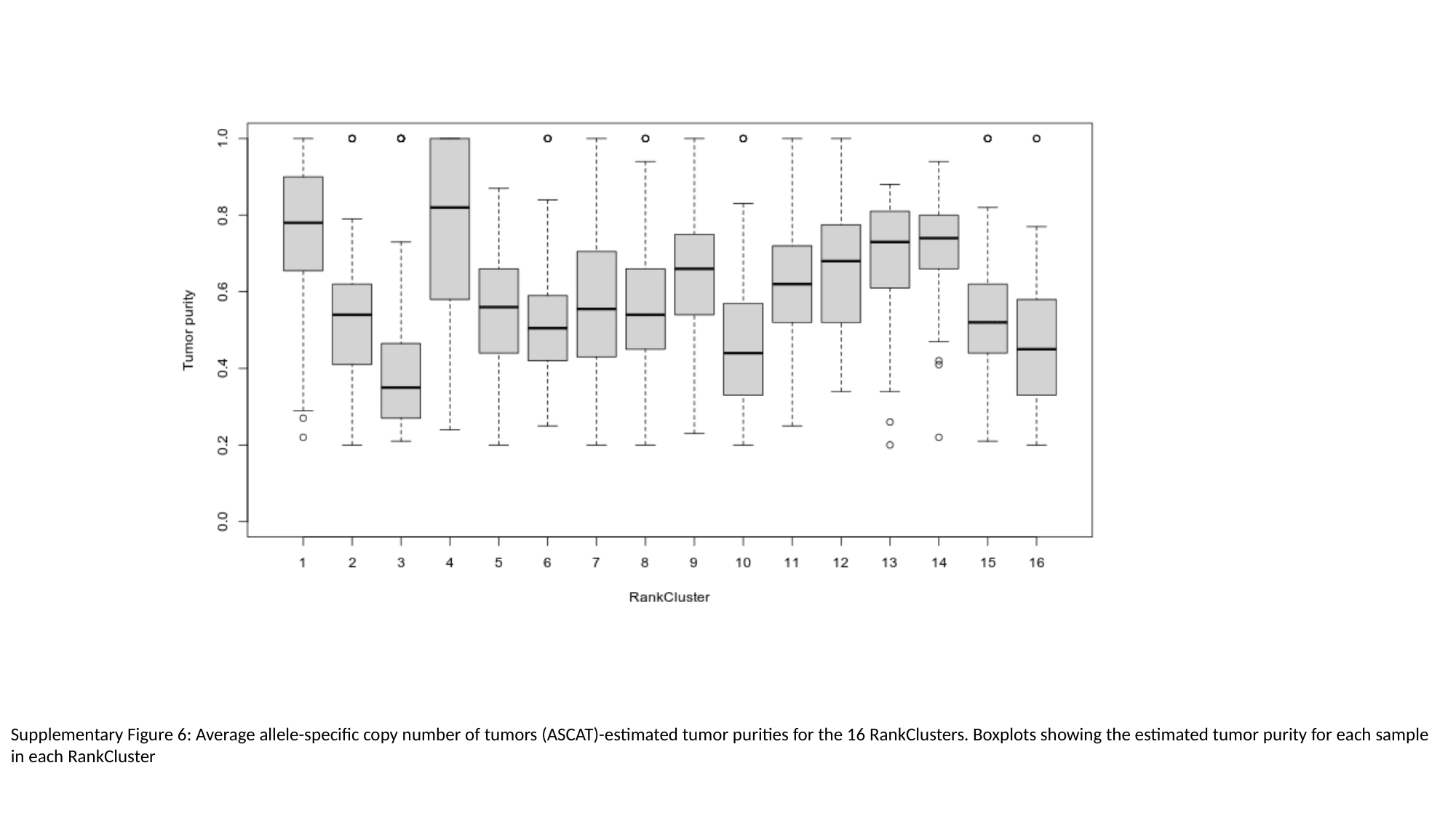

Supplementary Figure 6: Average allele-specific copy number of tumors (ASCAT)-estimated tumor purities for the 16 RankClusters. Boxplots showing the estimated tumor purity for each sample in each RankCluster
